# The Association Between Diabetes Mellitus During Pregnancy and Retinopathy of Prematurity

**DOI:** 10.3390/jcm15072790

**Published:** 2026-04-07

**Authors:** Lara Saaida, Eilon Shany, Ahed Imtirat, Nitzan Burrack, Victor Novack, Tamar Eshkoli

**Affiliations:** 1 Faculty of Health Sciences, Ben-Gurion University of the Negev, Beer-Sheva 84105, Israel; saaidalara97@gmail.com (L.S.); eilons@clalit.org.il (E.S.); amtirat@gmail.com (A.I.); victorno@clalit.org.il (V.N.); 2 Neonatal Intensive Care Unit, Soroka University Medical Center, Beer-Sheeva 84101, Israel; 3 Department of Ophthalmology, Soroka University Medical Center, Beer-Sheeva 84101, Israel; 4 Soroka Clinical Research Center, Soroka University Medical Center, Beer-Sheeva 84101, Israel; 5 Department of Obstetrics and Gynecology, Soroka University Medical Center, Beer-Sheeva 84101, Israel

**Keywords:** prematurity, diabetes mellitus, retinopathy, retinopathy of prematurity (ROP)

## Abstract

**Background/Objectives**: We aimed to evaluate the association between diabetes mellitus (DM) during pregnancy and retinopathy of prematurity (ROP) in preterm infants younger than 32 gestational weeks or infants with low birthweight (<1500 g). **Methods**: We conducted a retrospective nested case–control study of all premature infants who were born alive and survived the post-delivery hospitalization period in Soroka Medical Center, with either gestational age younger than 32 weeks or birthweight less than 1500 g, during the years 2013–2021. The infants were divided into two groups according to ROP status. Multivariable Generalized Estimating Equations (GEE) were used to analyze the association between ROP and DM, adjusting for potential confounders, including maternal age, diabetes type (GDM vs. pre-gestational DM), gestational age, birthweight (<1250 g), duration of oxygen supplementation, antenatal corticosteroid courses, and birth plurality. **Results**: During the study period, there were 881 pairs of women and newborns who met the inclusion criteria. The ROP group included 345 infants (39.1%). Twenty-two (6.4%) of the mothers in the ROP group were diagnosed with DM during pregnancy compared with 52 of 536 (9.7%) in the control group (*p* = 0.082). ROP was associated with oxygen treatment (OR 1.05; 95% CI, 1.03–1.08; *p* < 0.001), birthweight < 1250 g (OR 2.70; 95% CI, 1.93–3.78; *p* < 0.001) and advanced maternal age (OR 1.04; 95% CI, 1.01–1.06; *p* = 0.006). Prenatal steroid treatment was identified as a significant protective factor against ROP (OR 0.73; 95% CI, 0.60–0.89; *p* = 0.002). No statistically significant association was observed between maternal DM and ROP (OR 0.62; 95% CI 0.34–1.13; *p* = 0.12). These findings should be interpreted cautiously given the retrospective design and the limited availability of glycemic control data. **Conclusions**: Maternal diabetes mellitus was not significantly associated with the risk of ROP in this cohort.

## 1. Introduction

Diabetes mellitus (DM) during pregnancy is one of the most common morbidities among women in their reproductive age. In addition, DM rates are expected to increase even more due to the obesity epidemic [1]. DM during pregnancy includes both pregestational and gestational diabetes. In both cases, the mother and the fetus are exposed to a wide range of risks and complications during pregnancy [2]. In normal pregnancy, there is an increase in insulin resistance which causes compensatory mechanism of increasing insulin secretion from beta cells in the pancreas to keep normal blood glucose levels. Nevertheless, in instances of DM during pregnancy, this physiological mechanism appears to be ineffective, and insulin secretion becomes inadequate to compensate for the insulin resistance [3].

The WHO defines preterm birth as all births before 37 completed weeks of gestation. Surviving preterm infants are at risk of short- and long-term morbidities such as respiratory distress syndrome, necrotizing enterocolitis, retinopathy of prematurity (ROP), seizures, and other health problems [4,5]. These complications have a prominent global impact as a leading cause of mortality among children aged below 5 years [4], even though these complications are being observed in just 1–2% of all births [5].

Prior research has indicated that women with DM during pregnancy are at an increased risk for spontaneous preterm birth and as a result are at risk for increased prematurity complications [6,7]. While some reviews demonstrated a clear association between DM and prematurity complications such as ROP [6,7,8], other studies have reported no significant differences in mortality and morbidity when comparing infants born to diabetic mothers and those born to non-diabetic mothers [5,9,10].

Considering the rising prevalence of diabetes mellitus (DM) and its potential health burdens on both mothers and infants within medical systems, coupled with the scarcity of clinical data and inconclusive findings, there is a compelling need for further research.

The biological mechanisms linking maternal hyperglycemia to neonatal retinal vascular development are not fully understood. Chronic hyperglycemia has been shown to upregulate VEGF expression in retinal endothelial cells through oxidative stress pathways, potentially disrupting the tightly regulated angiogenic balance required for normal retinal vascularization [11]. Additionally, IGF-1 acts as a permissive factor enabling maximal VEGF-dependent retinal vessel growth, and low IGF-1 levels—as may occur in the setting of disrupted insulin signaling—have been shown to suppress normal retinal vascularization despite adequate VEGF expression [12].

We hypothesized that DM during pregnancy may influence the development of ROP in infants born prematurely.

The primary objective of our study was to compare the incidence of ROP between premature infants at risk of developing ROP born to mothers with DM during pregnancy versus those infants born to mothers without DM. Secondary objectives were to assess associations of ROP with medical interventions such as prenatal steroids treatment and oxygen treatment after delivery.

## 2. Materials and Methods

Study design: A nested case–control study was conducted within a cohort of premature infants admitted to the Neonatal Intensive Care Unit (NICU) of Soroka University Medical Center (SUMC) between 1 January 2013, and 31 December 2021. The study aimed to investigate the association between diabetes during pregnancy and the status of ROP in premature infants.

Study Population: The study population comprised infants who were members of the Clalit Health Maintenance Organization (CHS) in the Southern District of Israel and were born at SUMC between 2013 and 2021. Included in the study were infants born before 32 weeks of gestation or with a birthweight of less than 1500 g, as this population is at the highest risk for ROP and routinely undergoes ophthalmological screening per accepted clinical guidelines. A lower gestational age limit of 23 weeks was applied, as survival below this threshold is exceptionally rare and such infants represent a clinically distinct population whose inclusion would introduce significant heterogeneity into the cohort. Infants’ clinical information was coupled with their mothers’. Excluded were infants with unrecorded gestational age, those who were born before 23 weeks gestation, infants with chromosomal abnormalities or congenital abnormalities, and infants who died within seven days from birth.

Data Sources and Definitions: Data of Clalit members between 2013 and 2021 was collected from the SUMC computerized database by the Clinical Research Center of SUMC. Data of all preterm infants who met all inclusion criteria and none of the exclusion criteria was collected, including medical diagnosis and demographic characteristics for both infants and mothers. Records with incomplete charting of infant/mother matching were excluded. All diagnoses were classified according to registered diagnoses according to the International Classification of Diseases, 9th revision (ICD-9). We determined a diagnosis of ROP according to the registered ICD-9 code (362.20, 362.22–362.27) or free-text description in clinical records. In both cases, an attending pediatric ophthalmologist at SUMC made the diagnosis according to the accepted criteria. Maternal DM was classified into two subtypes: gestational diabetes mellitus (GDM), defined as glucose intolerance first identified during pregnancy, and pre-gestational diabetes (Type 1 or Type 2 DM), defined as a diagnosis of diabetes mellitus prior to conception. Analyses were performed both for any maternal DM as a combined exposure and stratified by subtype. Proxy measures of maternal glycemic management, including insulin use and oral hypoglycemic agent use during pregnancy, were extracted from clinical records where available. Maternal HbA1c values and longitudinal outpatient glucose profiles were not systematically recorded in the database and could not be included.

ROP stage was classified according to the International Classification of Retinopathy of Prematurity. Severe ROP was defined as stage 3 or higher.

Multiple births were not excluded from the analysis.

Statistical Analysis: Univariable analysis was used to compare sociodemographic and clinical characteristics according to the status of ROP of the premature infants. Social point score was determined using the Israel Central Bureau of Statistics (ICBS) classification of the residential SPS rank at the city/town/village level. Ranks are on a scale from one to 10, with lower ranks representing a lower SPS. This aggregate score is calculated using multiple socio-demographic and economic factors, including residents’ financial resources, housing conditions, motorization level, education and employment profile. Independent sample t-tests were used for continuous variables with a normal distribution (such as birthweight, gestational age, mother’s age, PH values and glucose levels), including mean and standard deviation. Wilcoxon rank-sum test was used to compare non-parametric values for count variables not meeting non-normal distribution assumptions (e.g., SPS, gravidity, Apgar score, Hospitalization duration, Mg sulfate doses, steroids doses and days of O_2_ treatment). Categorical variables were compared using Chi-square or Fisher exact test in variables with low-count data (such as maternal ethnicity, infant gender, diabetes status, IVF pregnancy, presence of pregnancy complications; preeclampsia, placenta accrete, placenta previa, PPROM and presence of infant’s morbidities; sepsis, IVH, NEC, BPD). To account for the potential non-independence of outcomes within multiple births, we used Generalized Estimating Equations (GEEs) with an exchangeable correlation structure to assess the association between maternal DM and ROP. Potential confounders were considered based on the univariable analysis and the predetermined clinical significance of the variables. Variables such as maternal age, gestational age, infant gender, birthweight (<1250 g), duration of oxygen supplementation, antenatal corticosteroid courses, birth plurality, and diabetes type (GDM, pre-gestational DM, or no diabetes) were included in the model. A 2-sided *p* < 0.05 was considered statistically significant and reported as a *p* value and 95% confidence interval. All models and statistical analyses were conducted and performed using R (version 4.2.0). All research activities were conducted following the Declaration of Helsinki and were approved by the local Institutional Review Board (IRB) (0151-21-SOR). Due to the retrospective nature of this study, no informed consent was required.

## 3. Results

**Figure 1:**
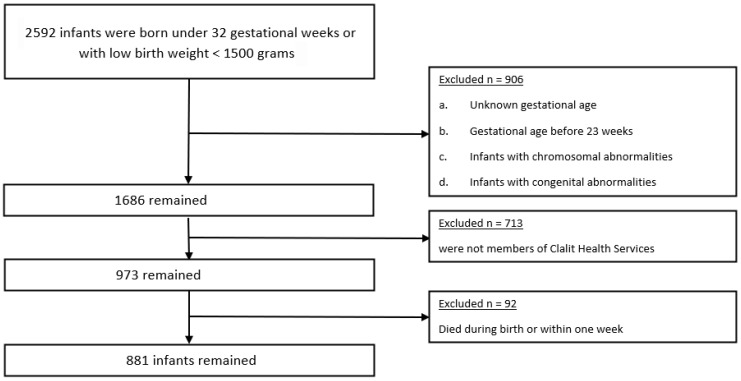
Exclusion criteria.

Throughout the study duration, 2592 infants were born to women with DM during pregnancy, either before 32 weeks of gestation or with a birthweight of less than 1500 g. We excluded 906 infants due to: unknown gestational age, less than 23 weeks gestation, chromosomal or congenital abnormalities. Of the 805 excluded infants, 713 infants were not members of Clalit Health Services and 92 infants died within one week of birth ( Figure 1).

Of the remaining 881 infant–mother pairs in our study, 345 infants (39.1%) were diagnosed with ROP. Of the 345 infants diagnosed with ROP, 338 (97.9%) had stage 0–2 and 7 (2.0%) had severe ROP (stage 3–5). Notably, no infant with severe ROP was born to a diabetic mother. Given the small number of severe ROP cases, multivariable analysis for this subgroup was not performed.

No significant differences were detected between the two groups regarding demographic characteristics of the ROP group vs. the non-ROP group (Table 1).

A comparison of pregnancy characteristics and complications between the two groups are presented in Table 2. No statistically significant difference in the prevalence of maternal DM was observed between the ROP and control groups (6.4% vs. 9.7%; *p* = 0.082), though the difference approaches statistical significance and should be interpreted cautiously. Proxy measures of glycemic management were available for a subset of diabetic mothers. Among mothers with diabetes, Insulin use was documented in 2 of 52 (3.8%) in the control group and 3 of 22 (14%) in the ROP group, and oral hypoglycemic use in 3 of 52 (5.8%) vs. 4 of 22 (18%). When stratified by diabetes subtype, no significant difference was observed between groups (*p* = 0.16; Table 2). Gestational DM was present in 35 mothers (6.5%) in the control group and 17 (4.9%) in the ROP group; pre-gestational DM was present in 17 (3.2%) and 5 (1.4%), respectively. In the multivariable GEE model, gestational DM (OR 0.80; 95% CI 0.40–1.60; *p* = 0.5) was not significantly associated with ROP. Pre-gestational DM showed a nominally significant inverse association (OR 0.32; 95% CI 0.11–0.91; *p* = 0.033); however, this result should be interpreted with caution given the very small number of pre-gestational DM cases (*n* = 22 total). Mothers of infants with ROP delivered at earlier gestational age (29.15 vs. 30.75 weeks, *p* < 0.001) and were less likely to receive any prenatal steroids (42% vs. 48%, *p* = 0.13).

Infant characteristics and interventions are shown in Table 3. Infants in the ROP group had a lower birthweight (1189 vs. 1396, *p* < 0.001) and lower Apgar scores at 1 and 5 min of age (7 vs. 8, *p* = 0.011 and 9 vs. 10, *p* = 0.002 respectively), had a longer duration of hospitalization (50.9 vs. 30.1 days; *p* < 0.001) and higher incidence of IVH (11% vs. 6.2%, *p* = 0.014) and BPD (10% vs. 1.9%, *p* < 0.001). Also, infants diagnosed with ROP were more likely to need oxygen therapy (94% vs. 77%, *p* < 0.001) and had overall a longer duration of oxygen therapy (4.63 vs. 15.41 days, *p* < 0.001) compared to the non-ROP group.

In the multivariable GEE model (Table 4), ROP was associated with longer duration of oxygen supplementation (OR 1.05; 95% CI 1.03–1.08; *p* < 0.001), birthweight less than 1250 g (OR 2.70; 95% CI 1.93–3.78; *p* < 0.001), and advanced maternal age (OR 1.04; 95% CI 1.01–1.06; *p* = 0.006), though the magnitude of this association was modest. Antenatal corticosteroid treatment (OR 0.73; 95% CI 0.60–0.89; *p* = 0.001) was identified as a protective factor. Birth plurality was not significantly associated with ROP (OR 1.26; 95% CI 0.82–1.95; *p* = 0.3). No statistically significant association between maternal diabetes and ROP was demonstrated (OR 0.62; 95% CI 0.34–1.13; *p* = 0.12).

## 4. Discussion

In the present study, maternal diabetes mellitus was not significantly associated with the risk of ROP in preterm infants, in either univariable or multivariable analyses. However, the difference in DM prevalence between groups (6.4% vs. 9.7%, *p* = 0.082) approaches statistical significance, and these findings should be interpreted with caution given the retrospective design and the limited availability of glycemic control data.

These findings are consistent with the study conducted by Persson M and colleagues [9], who carried out a large retrospective cohort study in 7 national networks in high-income countries involving 76,360 very preterm infants (24–31 weeks’ gestation) with a birthweight of less than 1500 g. Similar to our study, their research revealed no discernible association between maternal DM (MDM) and severe morbidity, including the occurrence of severe ROP, in preterm infants, with severe ROP rates of 4.8% in case group and 7.2% in control group. A Canadian-based study by Rehan and associates involving a double cohort on 582 very-low-birthweight infants (BW < 1500 g) found no association between ROP of any stage and maternal diabetes (30% in the diabetic group vs. 26% in the non-diabetic group) [13].

Conversely, a retrospective cohort study conducted in the United States by Opera CN [6] that included 883 maternal–neonatal pairs reported an association between DM during pregnancy and the development of ROP in neonates with very low birthweight (<1500 g). Opera CN et al. reported that the odds of an infant with severe ROP having a diabetic mother is 3.5 times higher compared to a non-diabetic mother. Additional retrospective Turkish study by Tunay ZO and colleagues [8], studied the relationship between MDM and ROP in infants with birthweight >1500 g. The study was conducted on 336 infants and reported an increased risk of ROP among infants born to diabetic mothers, with ROP rates of 78.2% in case group and 14.7% in control group.

The variations among the studies could be attributed to differences in study populations, designs, durations, diverse screening and diagnostic protocols, and, most significantly, variations in glycemic control.

Maternal hyperglycemia may influence fetal retinal vascular development through upregulation of VEGF expression and oxidative stress-mediated endothelial dysfunction [11]. Disrupted insulin-IGF-1 signaling in the hyperglycemic milieu may further impair retinal vascularization, as low IGF-1 levels have been shown to prevent normal VEGF-dependent vessel development in the immature retina [12]. Whether glycemic control during pregnancy modulates these pathways could not be assessed in the present study due to the unavailability of HbA1c data.

Furthermore, our data showed that birthweight < 1250 g, oxygen treatment and advanced maternal age are associated with a higher risk of developing ROP. Gestational age showed a non-significant trend toward reduced ROP risk, while steroid treatment was identified as a protective factor.

Our secondary findings are in line with the current known clinical factors associated with ROP. Thus, most of the studies on ROP have consistently identified either the degree of prematurity or the infant’s size as the most significant factors associated with the risk of ROP development [14,15,16,17].

Also, infants requiring ventilatory support typically face an elevated risk of developing ROP, and the longer oxygen therapy is administered, the greater the likelihood of ROP occurrence [13,15,16].

As for maternal age, Wu et al. [18] suggested that advanced maternal age is a significant risk factor, while other studies have presented divergent outcomes. These conflicting findings may be attributed to the substantial variability in maternal age ranges across the various studies.

The present study has several strengths. First, this is a relatively large single-center cohort of 881 preterm infant–mother pairs with detailed clinical data, providing adequate power to detect associations between maternal diabetes and ROP. Second, the retrospective design allowed for the inclusion of consecutive infants over a nine-year period, minimizing selection bias. Third, all ROP diagnoses were confirmed by an attending pediatric ophthalmologist, ensuring diagnostic accuracy. Finally, the multivariable GEE model accounted for established ROP risk factors including gestational age, birthweight, oxygen supplementation, and antenatal corticosteroids, as well as for the non-independence of multiple births.

Despite these strengths, the present study has several limitations. Although we performed a stratified analysis by diabetes subtype, the small number of pre-gestational DM cases (*n* = 22) substantially limits the statistical power of this comparison. Pre-gestational and gestational diabetes differ in their pathophysiology, timing of glycemic exposure, and associated neonatal risks, as pre-gestational diabetes may affect fetal development from the earliest weeks of gestation, whereas GDM typically manifests in the second or third trimester. Future studies with larger samples should address these subtypes separately.

Additionally, detailed glycemic control data—including maternal HbA1c levels and longitudinal glucose profiles—were unavailable in this retrospective dataset. Prior studies have demonstrated that parameters of carbohydrate metabolism, including HbA1c and fasting glucose, correlate with metabolic markers during pregnancy, underscoring the importance of glycemic characterization in obstetric research [19]. The absence of such data in our cohort represents a recognized limitation of retrospective administrative datasets. Also, modern continuous glucose monitoring (CGM) technologies may offer more granular glycemic data in future prospective studies [20]. Insulin and oral hypoglycemic use were documented in only a small subset of diabetic mothers and serve as imperfect proxies for overall glycemic management. Future prospective studies should incorporate longitudinal glycemic control data to more precisely characterize this exposure. Furthermore, only 7 infants had severe ROP (stage ≥ 3), precluding a meaningful analysis of risk factors specific to this subgroup. Prior studies have suggested that risk factors may differ by ROP severity; Gursoy et al. demonstrated that GDM was associated with treatment-requiring ROP and stage 3 disease, while pre-gestational diabetes was associated specifically with stage 3 ROP [21]. In our cohort, no infant with severe ROP was born to a diabetic mother, a finding that may partly explain the overall absence of a statistically significant association between maternal diabetes and ROP.

A further limitation is the exclusion of 92 infants who died within the first seven days of life. These infants represent the most critically ill subset and their exclusion may have introduced survivorship bias, potentially underestimating morbidity rates, including ROP risk, in the overall population of very preterm infants.

Finally, additional maternal variables such as gynecological comorbidities (e.g., PCOS, endometriosis) and personal or family medical history were not systematically collected in this retrospective dataset and could not be adjusted for in the analysis.

## 5. Conclusions

In this retrospective cohort of 881 preterm infants, maternal diabetes mellitus was not significantly associated with the risk of retinopathy of prematurity. The established risk factors for ROP—lower gestational age, lower birthweight, and prolonged oxygen supplementation—remained the dominant predictors in multivariable analysis. Future prospective studies incorporating detailed glycemic control data are needed to further characterize the relationship between maternal diabetes and neonatal retinal vascular outcomes.

## Figures and Tables

**Table 1:** Demographic characteristics of the study population (*N* = 881).

Characteristic	Control, *N* = 536 ^1^	ROP, *N* = 345 ^1^	*p*-Value
Maternal age (years)	28.59 (6.29)	29.40 (6.26)	0.062
Ethnicity			0.19
Arab	330 (62%)	197 (57%)	
Jewish	206 (38%)	148 (43%)	
Socioeconomic status score (1–5 scale)	4.02 (2.16)	4.13 (2.13)	0.40
(Missing)	132	75	
Infant sex			0.36
Female	270 (50%)	163 (47%)	
Male	266 (50%)	182 (53%)	

^1^ Mean (SD); *n* (%).

**Table 2:** Pregnancy characteristics and complications (*N* = 881).

Characteristic	Control, *N* = 5361	ROP, *N* = 3451	*p*-Value
Diabetes status			0.16
No diabetes	484 (90%)	323 (94%)	
Gestational DM	35 (6.5%)	17 (4.9%)	
Pre-gestational DM	17 (3.2%)	5 (1.4%)	
Gravidity	3.31 (3.38)	3.39 (2.78)	0.69
IVF conception	68 (13%)	45 (13%)	0.88
Gestational age (weeks)	30.75 (2.35)	29.15 (2.19)	<0.001
Antenatal corticosteroid courses			0.13
0	281 (52%)	199 (58%)	
≥1	255 (48%)	146 (42%)	
Antenatal magnesium sulfate courses	0.88 (1.85)	0.85 (1.85)	0.70
Birth plurality			0.96
Singleton	428 (80%)	276 (80%)	
Multiple	108 (20%)	69 (20%)	
Preeclampsia	103 (19%)	74 (21%)	0.42
Placenta accreta	5 (0.9%)	6 (1.7%)	0.36
Placenta previa	33 (6.2%)	26 (7.5%)	0.42
PPROM	135 (25%)	103 (30%)	0.13

*n* (%); Mean (SD).

**Table 3:** Infant Characteristics (*N* = 881).

Characteristic	Control, *N* = 5361	ROP, *N* = 3451	*p*-Value
Birthweight (g)	1396.69 (303.27)	1189.91 (293.25)	<0.001
Cord blood pH	7.29 (0.10)	7.29 (0.11)	0.96
(Missing)	51	28	
Apgar score, 1 min	8.00 (6.00, 9.00)	7.00 (5.00, 9.00)	0.011
Apgar score, 5 min	10.00 (9.00, 10.00)	9.00 (8.00, 10.00)	0.002
NICU length of stay (days)	30.10 (12.30)	50.94 (21.30)	<0.001
Sepsis	25 (4.7%)	15 (4.3%)	0.83
Intraventricular hemorrhage (IVH)	33 (6.2%)	37 (11%)	0.014
Necrotizing enterocolitis (NEC)	20 (3.7%)	15 (4.3%)	0.65
Bronchopulmonary dysplasia (BPD)	10 (1.9%)	35 (10%)	<0.001
Oxygen supplementation	411 (77%)	324 (94%)	<0.001
Duration of oxygen supplementation (days)	4.63 (7.08)	15.41 (17.89)	<0.001
Minimum glucose (mg/dL)	58.21 (10.10)	57.52 (9.85)	0.27
Maximum glucose (mg/dL)	149.34 (70.12)	158.57 (47.78)	<0.001
Mean glucose (mg/dL)	95.41 (22.93)	96.36 (15.60)	0.002

Mean (SD); Median (Q1, Q3); *n* (%).

**Table 4:** Multivariable GEE (Generalized Estimating Equations) model (*N* = 881).

Characteristic	OR	95% CI	*p*-Value
Maternal diabetes mellitus	0.62	0.34, 1.13	0.12
Birth plurality			
Singleton	—	—	
Multiple	1.26	0.82, 1.95	0.30
Maternal age (years)	1.04	1.01, 1.06	0.006
Birthweight <1250 g	2.70	1.93, 3.78	<0.001
Duration of oxygen supplementation (days)	1.05	1.03, 1.08	<0.001
Gestational age (weeks)	0.93	0.86, 1.02	0.12
Antenatal corticosteroid courses	0.73	0.60, 0.89	0.001

Abbreviations: CI = Confidence Interval, OR = Odds Ratio.

## Data Availability

The original contributions presented in this study are included in the article. Further inquiries can be directed to the corresponding author.

## Abbreviations

The following abbreviations are used in this manuscript:

DM	Diabetes Mellitus
ROP	Retinopathy of Prematurity
NICU	Neonatal Intensive Care Unit
SUMC	Soroka University Medical Center
CHS	Clalit Health Maintenance Organization
ICD9	International Classification of Diseases, 9th revision
ICBS	Israel Central Bureau of Statistics
